# The Effect of Resistance to Bt Corn on the Reproductive Output of *Spodoptera frugiperda* (Lepidoptera: Noctuidae)

**DOI:** 10.3390/insects13020196

**Published:** 2022-02-14

**Authors:** Natália de Souza Ribas, Jeremy N. McNeil, Hernane Dias Araújo, Bruna de Souza Ribas, Eraldo Lima

**Affiliations:** 1 Departamento de Entomologia, Universidade Federal de Viçosa, Viçosa 36570-900, Brazil; hernanearaujo@gmail.com (H.D.A.); bruna.ribas118@gmail.com (B.d.S.R.); eraldo.lima@ufv.br (E.L.); 2 Department of Biology, University of Western Ontario, London, ON N6A 5B7, Canada

**Keywords:** GMO, fall armyworm, spermatophore, host-plant resistance

## Abstract

**Simple Summary:**

The use of transgenic crops expressing insecticidal proteins from *Bacillus thuringiensis* (Bt) has been a successful strategy to control the fall armyworm (FAW) *Spodoptera frugiperda*. However, resistance to many Bt proteins has been observed, which could reduce the effectiveness of this approach as a control strategy. In this study, we compared the reproductive traits of susceptible and resistant FAW males and females to determine if there are costs associated with resistance. Our data provided clear evidence that the development of resistance to the Bt protein affected the reproductive capacity of resistant FAW males.

**Abstract:**

The fall armyworm (FAW) *Spodoptera frugiperda* is the most significant lepidopteran corn pest in South American countries. Transgenic Bt corn, producing the Cry1Fa toxins, has been used to control this pest, but there is clear evidence that some FAW populations have developed resistance. To determine if there are costs associated with resistance, we compared the mass of adults, the duration of mating, and the mass of the first spermatophore produced, as well as the lifetime fecundity and fertility of once-mated susceptible (SS) and resistant (RR) females. Adult mass was affected by both sex and strain, with SS females being significantly larger than RR ones, while the inverse was true for males. RR pairs took significantly longer to mate than SS pairs, yet the mass of spermatophores produced by RR males was significantly less than those of SS males. The total number of eggs laid did not differ but the fertility of eggs from once-mated RR pairs was significantly lower than that of SS pairs. Our data provided clear evidence that the development of Bt resistance affected the reproductive capacity of resistant FAW.

## 1. Introduction

The use of transgenic crops producing insecticidal proteins from *Bacillus thuringiensis* (Bt) has been a successful strategy for the control of important agricultural lepidopteran pests [1,2,3], including the fall armyworm (FAW) *Spodoptera frugiperda* (J. E. Smith, 1797) (Lepidoptera: Noctuidae), which is a global pest of corn [4,5,6,7,8,9,10,11]. However, resistance to most of the Bt proteins from the Cry1 group has been observed in numerous *S. frugiperda* populations in Puerto Rico, the United States, Brazil and Mexico [12,13,14,15]. Consequently, resistance could reduce the effectiveness of this approach as a control strategy. Although, if there is a fitness cost associated with resistance, susceptible individuals should do better in non-Bt areas (refuges), which may reduce the frequency of resistant alleles [16,17].

Bt-resistant insects have lower fecundity and fertility than those that are susceptible, as well as differences in the reproductive behavior and physiology of both sexes [16,18,19,20]. For example, in Lepidoptera, Bt-resistant females may spend less time calling and produce less pheromone [21], mate less frequently [22], or have a significant delay in ovary development [23]. It has been shown that resistant males may transfer less sperm than those that are susceptible [18] but, overall, less attention has been given to the effects of Bt resistance on males. 

During mating, male Lepidoptera transfer a spermatophore, which may vary in size and content due to several non-exclusive factors that include the mass of the male, age, past mating history, and the duration of mating [21,24,25,26]. In turn, the size of the spermatophore may influence female reproductive success, as larger ones generally contain more sperm and nutrients than smaller ones [27,28], resulting in females producing more eggs [29] with a higher incidence of hatching [30].

In this study, we used the fall armyworm, *S. frugiperda*, an important global pest of corn, to examine the effect of resistance to Cry1Fa toxin on the reproductive capacity of males and the impact of female reproductive output. To do this we compared (i) the mass of adults; (ii) the duration of mating; (iii) the mass of the first spermatophore produced; and (iv) the lifetime fecundity and fertility of susceptible and resistant pairs. The results indicate there are potential costs to reproduction associated with the development of resistance in the FAW, which would need to be considered when planning effective management programs against this important pest species.

## 2. Materials and Methods

### 2.1. Insect Rearing

The susceptible and resistant strains of the FAW came from the colony established from field-collected material with a final resistance ratio >185, and reared on Cry1Fa corn leaves every fifth generation to retain a level of resistance that would result in at least 50% mortality of susceptible larvae [31,32]. A minimum of 200 randomly selected adults from each colony were used at each generation. Our colonies were maintained at 27 ± 1 °C, 70 ± 15% R. H. under a 14L:10D photoperiod, as were all subsequent experiments. Adults were held in PVC (polyvinyl chloride) cages (30 cm high × 20 cm diameter), with sulfite paper on the inner walls for oviposition, as well as an ad libitum food source of 10% sugar/1% ascorbic acid solution that was changed every two days. Larvae were reared on an artificial diet [33], in groups as neonates and then reared individually from the 2nd instar in 16-cell PVC trays (Advento do Brasil Ind. e Comércio de Plásticos Ltd., Diadema, São Paulo, Brazil) until pupation. The pupae were sexed, with males and females subsequently held separately in square acrylic cages (30 cm × 30 cm × 30 cm). Every day, newly emerged moths were collected at the end of the photophase, chilled for 3 min to decrease activity and then weighed (Shimadzu AUW220D balance, Schimadzu Corporation, Kyoto, Japan). Each adult was held until needed in an individual 70 mL plastic container that had a cheesecloth top to allow air circulation, and provided 10% sugar solution ad libitum.

### 2.2. Bioassays 

A minimum of 10 3-day-old RR and SS pairs were used from each successive generation (generations 16–19) to ensure that differences between the SS and RR existed across generations. The pairs were set up in individual cages and observed throughout the scotophase and the duration of mating recorded. After mating, the females were held in individual 75 mL PVC cages internally coated with sulfite paper as an oviposition substrate and provided an ad libitum 10% sugar solution. The total number of eggs produced by each female and the number hatching were counted with the aid of a Leica EZ4 HD stereomicroscope.

An additional 10–13 pairs of 3-day-old RR and SS moths for each generation were set up and, immediately after mating had finished, each female was dissected and the spermatophore removed. Once dried on paper toweling, the spermatophores were weighed with an analytical balance precision of 0.1 mg (Shimadzu AUW220D, Schimadzu Corporation, Kyoto, Japan).

### 2.3. Statistical Analyses

Statistical analyses were performed in R (v. 4.0.0; R Development Core Team, 2020) using Analysis of Deviance (ANODEV; a maximum likelihood equivalent of ANOVA), followed by residual analysis to verify the suitability of distributions of the tested models. The effects of genotype and generation on total fecundity, fertility (eggs hatched/eggs laid), spermatophore mass, and adult mass were determined using generalized linear models (GLM) with Poisson, Binomial, and Gaussian distribution of errors, respectively. Finally, the effect of genotype and generation on mating duration was determined using survival analysis with Weibull distribution. The Least Squares Means (“emmeans” package) evaluated differences between treatments.

## 3. Results

**Figure 1:**
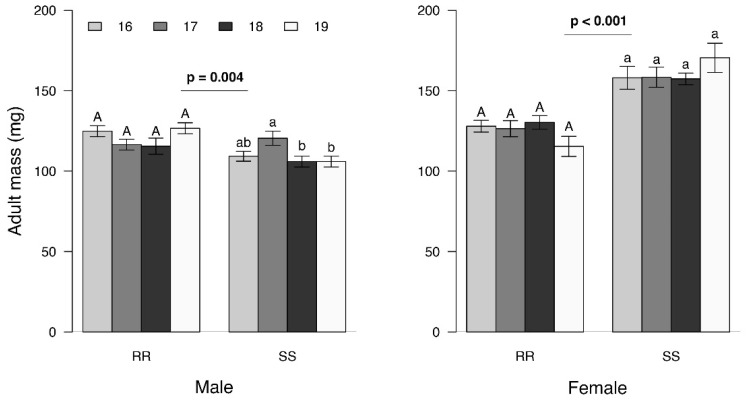
The mean ± SEM mass of resistant and susceptible female and male *S. frugiperda* adults in four different generations (*n* = 11–12 per treatment). The values *p* = 0.004 and *p* < 0.001 represent the difference between the RR and SS genotypes, and the letters represent the differences between the generations (uppercase for differences between RR genotype and lowercase for differences between SS genotype). FDR-corrected *p* values are given for treatment comparisons (generalized linear model (family, gaussian)), followed by pairwise comparisons of Least Squares Means (LSM).

Adult mass was significantly affected by sex, genotype and generation (Figure 1; F_(3, 184)_ = 4.40; *p* = 0.005). SS females were heavier than SS males (*p* < 0.001) but there was no difference between RR males and RR females (*p* = 0.24). While RR males were heavier than SS males (*p* = 0.004), SS females were heavier than RR females (*p* < 0.001). There were no generational differences for SS females or both sexes of RR individuals (*p* > 0.05), but the mass of 17th generation SS males was greater than that of the 18th and 19th generations (*p* = 0.023).

**Figure 2:**
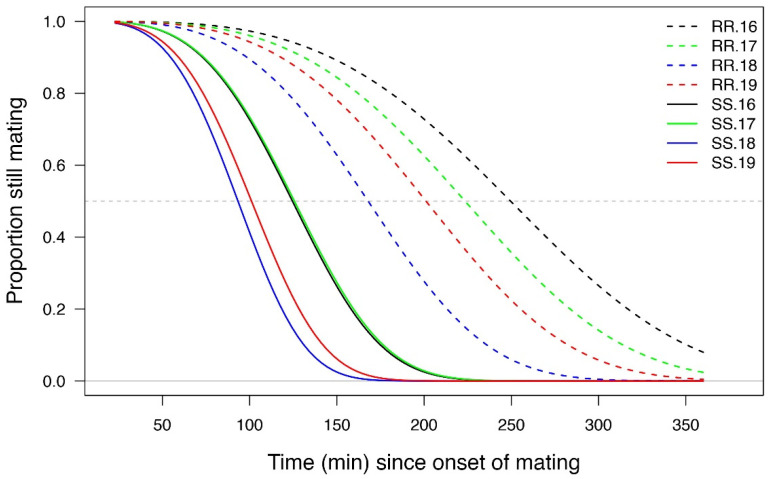
The proportion of resistant (RR) and susceptible (SS) *S. frugiperda* pairs from successive generations remaining in copula as a function of time since the onset of mating (*n* = 50 per genotype and *n* = 12–13 per generation). RR.16 and SS.16 = FAW from the 16th generation; RR.17 and SS.17 = FAW from the 17th generation; RR.18 and SS.18 = FAW from the 18th generation; and RR.19 SS.19 = FAW from 19th generation. Lognormal survival analysis (*p* < 0.001) followed by pairwise comparisons of Least Squares Means (LSM).

There was a significant effect of genotype in the mating duration (χ2 = 1089.3; *p* < 0.001), being longer for RR than SS pairs. In addition, there was an overall generation effect (χ2 = 1077.8; *p* = 0.009), with the duration of mating for both SS and RR pairs being longer for the 16th than for the 18th generation (*p* = 0.05); there were no differences between the other generations (*p* > 0.05) (Figure 2). However, for any given generation, RR pairs took significantly longer than their SS counterparts (*p* < 0.001).

**Figure 3:**
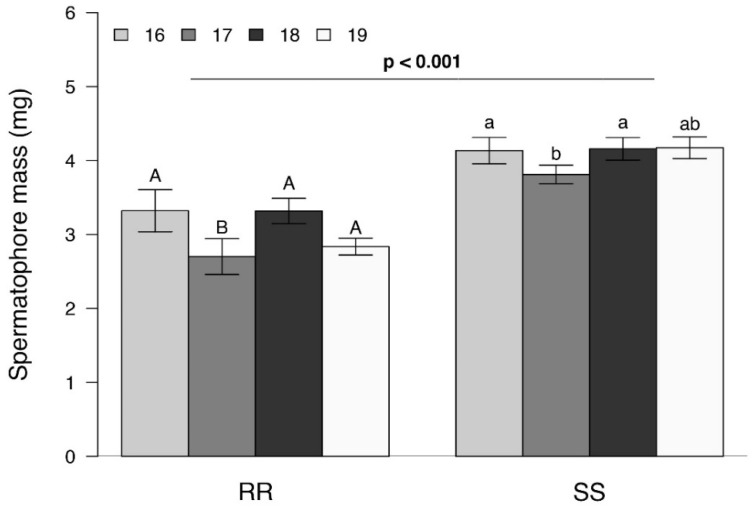
Mean ± SEM mass of spermatophores (mg) produced by resistant (RR) and susceptible (SS) *S. frugiperda* males when mated with females of the same genotype using four different generations (*n* = 50 per genotype and *n* = 12–13 per generation). FDR-corrected *p* values are given for treatment comparisons (generalized linear model (family, gaussian)), followed by pairwise comparisons of Least Squares Means (LSM). The value of *p* < 0.001 represents the difference between the RR and SS genotypes, and the letters represent the differences between the generations (uppercase for differences between RR genotype and lowercase for differences between SS genotype).

Overall, the mass of spermatophores produced by RR males was significantly lighter than those of SS males (F_(1, 98)_ = 63.81; *p* < 0.001), a difference that was consistent across generations (F_(3, 95)_ = 3.04; *p* = 0.033) (Figure 3). As seen in Figure 3, there was some degree of intergenerational variability within both SS and RR strains.

**Figure 4:**
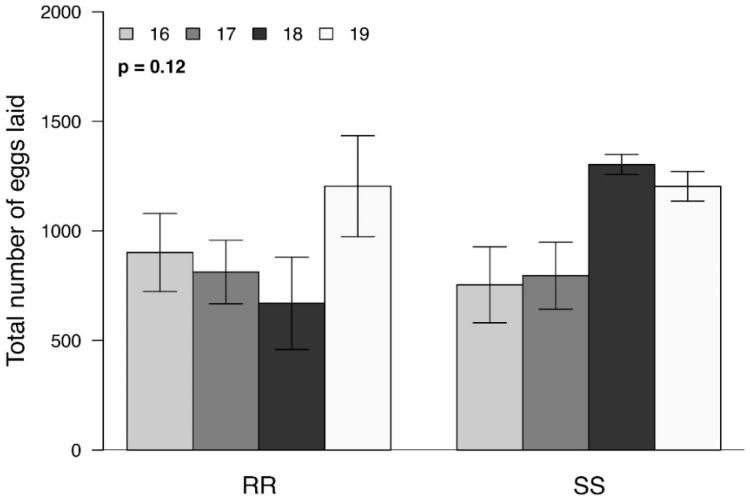
Mean ± SEM total number of eggs produced by resistant (RR) and susceptible (SS) *S. frugiperda* pairs (*n* = 46 per treatment) using four different generations (*n* = 11–12 per treatment). FDR-corrected *p* values are given for treatment comparisons (generalized linear model (family, Quasipoisson)), followed by pairwise comparisons of Least Squares Means (LSM). The value of *p* = 0.12 represents the result of the total model.

**Figure 5:**
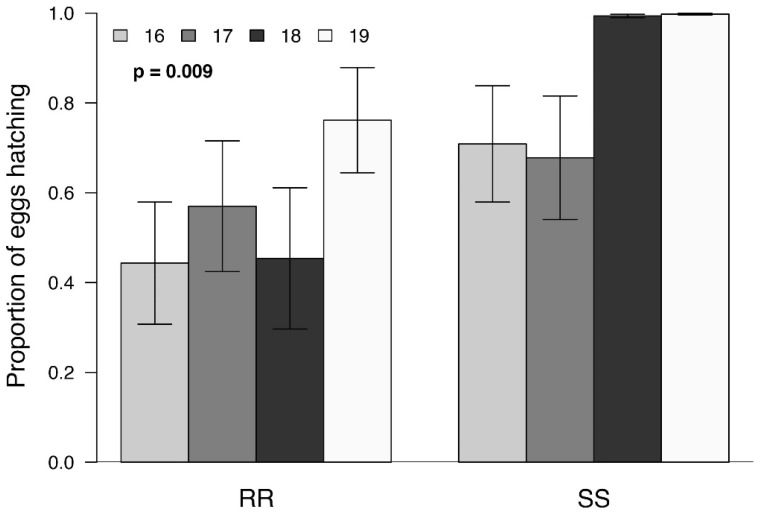
Mean ± SEM proportion of eggs produced by resistant (RR) and susceptible (SS) *S. frugiperda* females that hatched using four different generations (*n* = 46 mated egg-laying females per genotype and 11–12 per generation). FDR-corrected *p* values are given for treatment comparisons (generalized linear model (family, Quasibinomial)), followed by pairwise comparisons of Least Squares Means (LSM). The value of *p* = 0.009 represents the difference between the RR and SS genotypes; the generation did not affect the proportion of eggs hatching.

Overall, the mean lifetime fecundity of SS and RR pairs did not differ significantly (F_(3, 84)_ = 2.01; *p* = 0.12; Figure 4), but the fertility of the RR eggs was significantly lower than SS eggs (F_(1, 90)_ = 7.17; *p* = 0.009; Figure 5). There was no significant generational effect on either fecundity (F_(3, 87)_ = 2.26; *p* = 0.087) or fertility (F_(3, 87)_ = 2.57; *p* = 0.059).

## 4. Discussion

Santos-Amaya et al. [31] reported no apparent fitness cost between control and Cry1Fa-resistant fall armyworm lines over seven generations when comparing growth rate, larval stage, fecundity and fertility. However, the results of our experiments show that there can be a cost to RR pairs, findings that agree with other studies reporting fitness-related costs associated with resistance to Cry1Fa protein in the fall armyworm [34,35,36]. We did observe an inter-generational effect of resistance on the different reproductive parameter measures, which, to the best of our knowledge, has not been reported in other studies comparing Bt-susceptible and Bt-resistant insects. While there were no consistent trends, this could be a line of future research examining the extent to which differences occur in nature and the potential impact this might have on the level of resistance under field conditions.

Our results suggest that the effects observed are associated with the males. While RR males were significantly larger than their SS counterparts (Figure 1) and remained in copula for a significantly longer time (Figure 2), they produced significantly smaller spermatophores (Figure 3). While there is little evidence that the duration of mating affected spermatophore size [37,38], one would have expected RR males to produce larger spermatophores, given that a positive correlation between male mass and spermatophore size has been observed in a number of Lepidoptera [24,38,39]. The reduction in spermatophore size did not result in a decrease in the total number of eggs laid by RR females (Figure 4) but there was a significant reduction in the proportion of hatching (Figure 5). Additional research will be required to determine to what extent this is due to the number and/or quality of sperm and male accessory gland secretions produced, as all of these parameters have been shown to affect fertility in other species [22,26,27,29,40]. However, one cannot eliminate possible female effects relating to the transfer of sperm from the bursa copulatrix to the spermatheca [41,42,43] or the fertilization of eggs during oviposition [23].

These questions could be addressed by evaluating the reproductive output of pairs where a resistant male or female was mated with a susceptible individual. This would also provide insight into the overall effects that the cost of resistance might have on population numbers, especially as a certain level of assortative mating appears to exist under field conditions (unpublished data, Jeremy McNeil). Furthermore, if extended mating is uniquely associated with RR males, this could result in higher levels of predation [44,45,46].

## Data Availability

The datasets generated and/or analyzed during the current study are available from the corresponding authors on reasonable request.
